# Estimating dyskinesia severity in Parkinson’s disease by using a waist-worn sensor: concurrent validity study

**DOI:** 10.1038/s41598-019-49798-3

**Published:** 2019-09-17

**Authors:** Alejandro Rodríguez-Molinero, Carlos Pérez-López, Albert Samà, Daniel Rodríguez-Martín, Sheila Alcaine, Berta Mestre, Paola Quispe, Benedetta Giuliani, Gabriel Vainstein, Patrick Browne, Dean Sweeney, Leo R. Quinlan, J. Manuel Moreno Arostegui, Àngels Bayes, Hadas Lewy, Alberto Costa, Roberta Annicchiarico, Timothy Counihan, Gearòid Ò. Laighin, Joan Cabestany

**Affiliations:** 1 Consorci Sanitari de l’Alt Penedès i Garraf, Vilafranca del Pendès, Spain; 2 Electrical & Electronic Engineering Department, NUI Galway, Galway, Ireland; 3 Technical Research Centre for Dependency Care and Autonomous Living(CETpD), Universitat Politècnica de Catalunya, Vilanova i la Geltru, Spain; 4 Sense4Care, Cornellà de Llobregat, Spain; 5 Unidad de Parkinson y trastornos del movimiento (UParkinson), Centro Médico Teknon, Barcelona, Spain; 6 IRCCS Fondazione Santa Lucia, Rome, Italy; 7 Maccabi Healthcare Services, Tel Aviv, Israel; 8 School of Medicine, NUI Galway, Galway, Ireland; 9 Niccolò Cusano University of Rome, Rome, Italy; 10 Neurology Department, University Hospital Galway, Galway, Ireland; 11 School of Nursing and Midwifery, NUI Galway, Galway, Ireland

**Keywords:** Learning algorithms, Drug regulation, Drug delivery, Parkinson's disease, Parkinson's disease

## Abstract

Our research team previously developed an accelerometry-based device, which can be worn on the waist during daily life activities and detects the occurrence of dyskinesia in patients with Parkinson’s disease. The goal of this study was to analyze the magnitude of correlation between the numeric output of the device algorithm and the results of the Unified Dyskinesia Rating Scale (UDysRS), administered by a physician. In this study, 13 Parkinson’s patients, who were symptomatic with dyskinesias, were monitored with the device at home, for an average period of 30 minutes, while performing normal daily life activities. Each patient’s activity was simultaneously video-recorded. A physician was in charge of reviewing the recorded videos and determining the severity of dyskinesia through the UDysRS for every patient. The sensor device yielded only one value for dyskinesia severity, which was calculated by averaging the recorded device readings. Correlation between the results of physician’s assessment and the sensor output was analyzed with the Spearman’s correlation coefficient. The correlation coefficient between the sensor output and UDysRS result was 0.70 (CI 95%: 0.33–0.88; p = 0.01). Since the sensor was located on the waist, the correlation between the sensor output and the results of the trunk and legs scale sub-items was calculated: 0.91 (CI 95% 0.76–0.97: p < 0.001). The conclusion is that the magnitude of dyskinesia, as measured by the tested device, presented good correlation with that observed by a physician.

## Introduction

Parkinson’s disease (PD) is due to death of dopamine-producing neurons in the brain’s basal ganglia and is characterized by slowness of movement (bradykinesia), together with stiffness and postural instability, sometimes also with tremor^1^. Mild PD responds well to a treatment with L-Dopa and dopaminergic agonists. However, as the disease progresses, drug effects wane or last for a shorter time (*wearing-off*), which requires medication dosage adjustments in order to keep the symptoms under control throughout the day^2^. In spite of this, most patients develop motor fluctuations after 10 years. Patients, who have motor fluctuations switch between so-called “Off”-periods – when medication is ineffective and movement is difficult – and so-called “On”-periods – when medication is optimally effective and movement is fluid^3^. Additionally, on the transition between both states (On/Off), or during the period of maximum medication effect, patients may present dyskinesias, namely involuntary head, trunk or limb movements, which may even interfere with their activity^4^.

Dyskinesias are a consequence of the dopaminergic treatment, and in many cases they can be improved by adjusting the therapeutic schedule. This is difficult for physicians to do, as they are fluctuating symptoms, which appear and disappear throughout the daytime, with a hard-to-establish chronology. Currently, to obtain detailed information on the time sequence of these symptoms, physicians ask patients to keep written records of the times of the day when dyskinesias occur (patient diaries). However, these records also have their limitations, as dyskinesia consists of involuntary movements, mostly unperceived by the patient, who often does not recognize the moment when the symptom occurs, or forgets to record it. Also, the patient adherence to the method is poor, since recording the symptom timeline is a hard task, difficult to complete beyond a few days^5^. Therefore, an automatic monitoring system, capable of recording the timeline of the symptoms would be welcome by both the physicians and patients^4^.

Over the past decade, our research team has been developing a waist worn wearable monitor that detects several Parkinson’s symptoms and analyses their evolution over time.

In particular, the device can detect motor fluctuations^6,7^, bradykinesia^8^, freezing of gait and dyskinesia^9^; although, dyskinesia is detected in a dichotomous way – namely only its occurrence or not is detected at every moment, without information on its severity^10^. However, the severity of dyskinesia is a useful parameter. In severe dyskinesias a therapeutic action is needed, while in milder ones, which do not interfere with the patient’s activity, therapeutic adjustments are not necessary. Actually, although the algorithm developed was validated for dichotomous results, it produces a continuous numerical value whose magnitude is potentially related to the symptom severity and may therefore be useful for clinicians. This study aims at verifying or rejecting the hypothesis that the numerical output of the dyskinesia algorithm is correlated with the severity of dyskinesia, as measured with a clinical scale.

## Methods

This is a concurrent validity study comparing the output of a dyskinesia-detection algorithm, based on accelerometry, with the results of certain subscales of the Unified Dyskinesia Rating Scale (UDysRS)^11^.

In this study – which is a part of the MoMoPa-III project (Mobility Monitorization of Parkinson patients for therapeutic purposes - DTS15/00209 & INV_A088_P) –the inertial signals and video-record database from the earlier conducted REMPARK project (Personal Health Device for Remote and Autonomous Management of Parkinson’s Disease)^12^ were used. The REMPARK database was built with the aim of recording inertial signals corresponding to different motor symptoms of the Parkinson’s disease: bradykinesia, dyskinesia and freezing of gait. The sensor used for recording the REMPARK database was fully developed by the Technical Research Centre for Dependency Care and Autonomous Living (CETpD); this sensor records triaxial information, from 3 integrated inertial sensors (gyroscope, magnetometer and accelerometer), on a microSD card with a 200/second sampling frequency.^13^

The methods used to build the REMPARK database and their rationale, are described elsewhere^10,14^. Briefly, the database was built using a sample of 75 patients with idiopathic Parkinson’s disease, according to the UK Parkinson’s Disease Society Brain Bank clinical criteria^1^. Included patients were at least in a moderate phase of the disease (Hoehn and Yahr scale >2^15^) and presented motor fluctuations. Patients older than 80 years, patients with gait disorders of a cause other than Parkinson, patients with dementia and patients with implanted electronic devices were excluded. Patients were selected by convenience sampling among those managed by neurologists in four hospitals: Centro Médico Teknon (Spain), Fondazione Santa Lucia (Italy), Maccabi Healthcare Services (Israel) and University Hospital Galway (Ireland). To create the database, ambulatory measures were made, at the patients’ home and nearby areas outdoors, using the inertial sensor located on the left side of patients’ waist. The data collection protocol was designed to capture dyskinesias, freezing of gait, and motor fluctuations while walking around their home and outdoors (free monitoring in real ambient conditions). The protocol also included specific daily movements that could be mistaken with Parkinson symptoms, and therefore could cause false positive detections to occur: brushing teeth, drying a glass, cleaning a window or a piece of furniture, typing on the computer and cautiously carrying a glass of water from one room to other. In addition, the system records the time periods when patients were sitting or standing while waiting for instruction from the researchers. Afterwards, every accelerometer signal segment was identified and tagged according to the corresponding activity and symptoms; using the corresponding video recording which was synchronized with the sensor inertial signal. In the particular case of dyskinesias, the associated identification labels included references to the body segment involved (head, right leg, left leg, left arm, right arm, trunk) and their severity classified as mild or strong according to the opinion of the medical staff reviewing the video records. Besides inertial signals and synchronized video records, the database included sociodemographic variables (sex, age) and variables related to the Parkinson’s disease for every included patient: year of diagnosis, severity measured with the Hoehn & Yahr scale (H&Y)^15^, therapeutic schedule, Freezing of Gait Questionnaire (FOG-Q) score^16^ and scores of the Unified Parkinson’s Disease Rating Scale (UPDRS) on and off state^17^.

The Consoric Sanitari del Mresme Ethical Committee approved the research protocol for creating the REMPARK database. All participants signed an informed consent form before their inclusion in the study. All experiments were performed in accordance with relevant guidelines and regulations.

For the concurrent validity analysis presented in this article, patients presenting dyskinesias from the above described database were selected, excluding patients with only mild limb dyskinesia. The reason for excluding them is that in video records such dyskinesias are hard to distinguish from voluntary movements, and in some cases from tremor; thus, a good reference standard cannot be established for them. The rest of the patients with dyskinesias were included in the analysis. For the present study, a physician experienced in movement disorders reviewed the database video records of each patient (which lasted approximately for 30 minutes each) and assessed the severity of dyskinesias with the Part 3 of the UDysRS (Objective evaluation of dyskinesia disability: Intensity scale), which rates from 0 to 4 the intensity of the dyskinesia of 7 different body segments. The use of the UDysRS was licensed by the International Parkinson and Movement Disorder Society, and the physician received training in scale scoring, according to the instrument guidelines. The physician applied the scale at all times on the video, when he considered the patients presented dyskinesias. The UDysRS scores for the different video scenes were averaged to yield only one dyskinesia score for every patient. Also, the dyskinesia detection algorithm was applied to the inertial signals corresponding to each dyskinesia video scene rated by the physician and the results averaged to obtain a single value per patient. Due to privacy reasons, the participants’ faces were not recorded in many videos, thus the face dyskinesia item on the scale was not assessed or included in the analysis. The expert who reviewed the video-records had no access to the algorithm information.

The dyskinesia algorithm is based on frequency analysis and on the principle that low-frequency low-amplitude harmonics appear in movement inertial records of patients with dyskinesia^10^. According to this principle, while the patient is still (while sitting or standing up) characteristic harmonics appear in the signals, which are due to dyskinesia. In a simple way, the method considers the power spectrum in the frequency band, which is composed of harmonics 1–4 Hz – called the dyskinesia band – to detect dyskinesia. Furthermore, several more conditions are considered, which allow for better contextualization of a patient’s movements and consequently improve algorithm specificity. This article is focused on the lowest level of the algorithm previously published^10^, which analyses the signal’s 50% overlapped windows. The window’s length was selected on the basis of three key factors: the lowest limit of the frequency bands of interest – in this case 1 Hz – the sampling frequency – fixed at 40 Hz – and finally, the use of temporal values which are a power of two, a technical requirement to be able to use fast conversion Fast Fourier Transform (FFT) algorithms. Taking these three factors into account, 3.2 second-windows, or 128 sample-windows, were the most suitable choice. Thus, a characteristic value per window was obtained every 1.6 seconds, which resulted from the addition of the power spectrum of the harmonics associated to the dyskinesia band.

**Figure 1:**
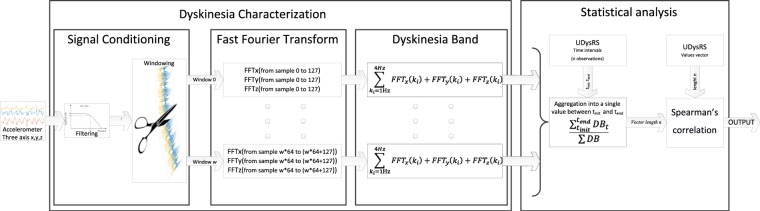
Schematic representation of the signal treatment and the statistical analysis process.

In summary, the accelerometer measurements are divided into windows of 3.2 seconds (50% overlapping) and the only characteristic used is the sum of the power spectrum in the dyskinesia frequency band of the three axes. An average is calculated from the vector, of this characteristic, incorporating the multiple relevant windows evaluated (the evaluations are only carried out when the patient is still). For example, if the physician has performed an evaluation of the UDysRS in a period comprised between the 120 and 240 seconds of a video, the output value of the dyskinesia algorithm is the average of the characteristics from the vector comprised between these seconds where the patient is still. In Fig. 1, a schematic representation of the data analysis is presented.

In the statistical analysis, the numerical value from the dyskinesia algorithm was compared with the UDysRS scale value by using the Spearman’s correlation coefficient. Every patient contributed to the calculation of this correlation coefficient with only one sensor output (the mean characteristic value from the windows included in the scenes rated by the physician) and only one UDysRS score (the mean of the physician’s scores). Correlation was first calculated with the total available UDysRS scores and, subsequently, using only with the trunk and lower-limb items since, given the sensor’s location on the body, it can be hypothesized that the correlation with such body segments would be better. The confidence interval for Spearman correlation was calculated by applying the Fisher transformation^18^.

The datasets generated and analyzed during the current study are available from the corresponding author on reasonable request.

## Results

The study included 13 patients, of which 8 were labeled in the database as with “mild trunk dyskinesias”, 3 were labeled as “severe trunk dyskinesias” and 2 patients as “severe limb dyskinesias”. Table 1 shows the demographic and clinical characteristics of included patients. A total of 6 hours of video (mean of 28 minutes per patient) were analyzed by the expert clinician. On average, the UDysRS scale was administered by the expert observer 7 times per video record.

**Table 1:** Demographic and health data of the participants.

	Age	Sex	H&Y^a^	% of the day in OFF^b^	FOG-Q^c^	Years from diagnosis	Dopamin total daily dose (mg)	UPDRS^d^ Off	UPDRS^d^ On
1	77	Male	2.5	<25	13	27	700	39	17
2	75	Male	3.0	<25	14	16	600	34	20
3	65	Male	3.0	25–50	14	8	650	43	16
4	60	Male	2.5	25–50	17	17	800	23	5
5	66	Male	4.0	50–75	17	11	900	38	8
6	73	Male	2.5	<25	9		500	26	
8	74	Female	3.0	<25	11	27	625	65	33
9	65	Female	3.0	25–50	20	18	825	56	15
10	74	Female	2.5	<25	20	26	500	48	16
11	71	Female	3.0	<25	16	11	875	27	10
12	73	Female	3.0	<25	13	11	1200	49	16
13	67	Male	3.0	25–50	14	24	1100	44	5
**Average**	70		2.9	38	15	18	773	41	15

^a^Hoehn & Yahr scale.

^b^As reported in the Unified Parkinson’s Disease Rating Scale.

^c^Freezing of Gait Questionnaire.

^d^Unified Parkinson’s Disease Rating Scale.

*Basal data from participant number 7 were unavailable in the database.

**Figure 2:**
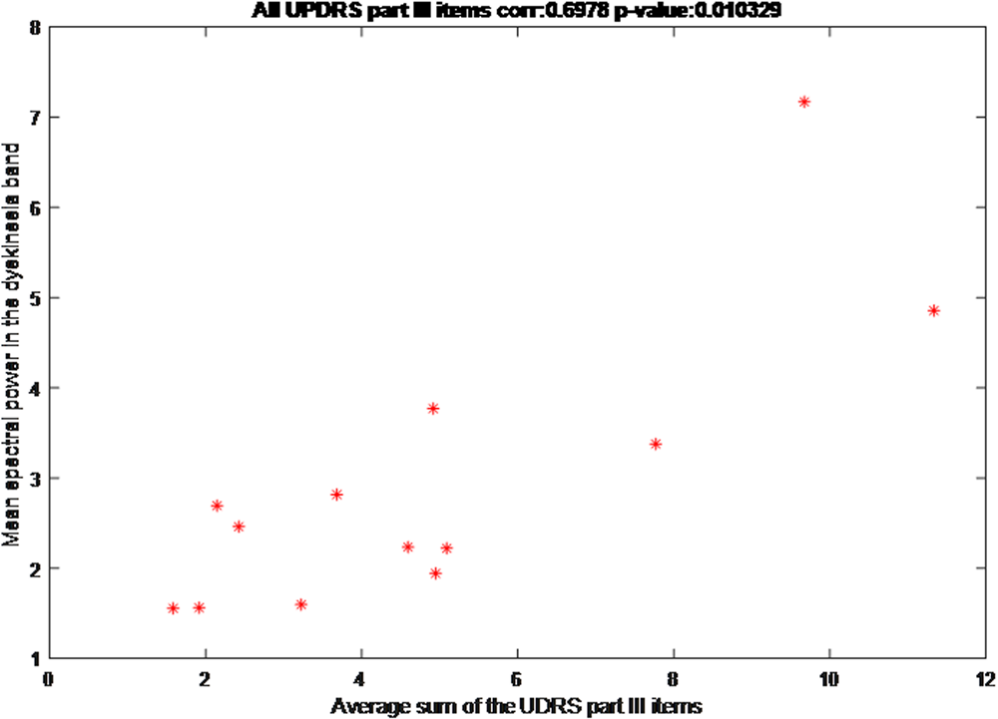
Correlation of total Unified Dyskinesia Rating Scale score with sensor output.

**Figure 3:**
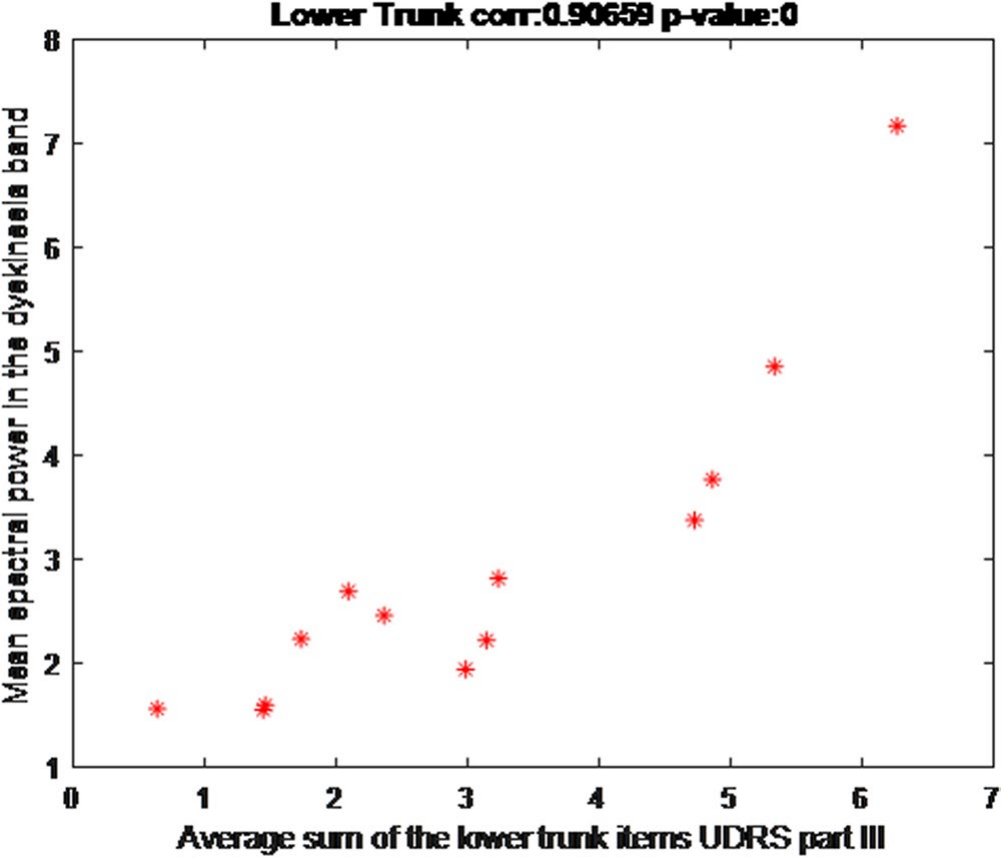
Correlation of lower-limb Unified Dyskinesia Rating Scale scores with sensor output.

The correlation between the mean UDysRS score for each patient and the sensor output was 0.70 (CI 95%: 0.33-0.88; p = 0.01). When only the mean trunk and lower-limb UDysRS scores from each patient were used, the correlation with the sensor output increased to 0.91 (CI 95% 0.76–0.97: p < 0.001). Figures 2 and 3 illustrate the correlation results. Table 2 details the correlation coefficient for every analyzed scale item.

**Table 2:** Spearman’s correlation between the dyskinesia algorithm and the Unified Dyskinesia Rating Scale.

Item	Ro	p-value
1.- Face	NaN	NaN
2.- Neck	0.39	0.396
3.- Right arm	0.25	0.430
4.- Left arm	0.53	0.148
5.- Trunk	0.64	**0**.**021**
6.- Right leg	0.77	**0**.**014**
7.- Left leg	0.70	**0**.**021**
Items: 5 + 6 + 7	0.91	<**0**.**001**
Total	0.70	**0**.**010**

Bold type: statistically significant finding.

## Discussion

In this study, the output of a dyskinesia-detection algorithm, previously developed by the authors, was found to be well correlated with the UDysRS scale (objective evaluation of dyskinesia disability: Intensity scale), specifically with the items related to trunk and lower-limb dyskinesias.

Experimental work has been done regarding detection of dyskinesias with inertial sensors (occurrence vs. non-occurrence of dyskinesia), which have been previously discussed by us^10^. However, few studies have addressed detection of the severity of dyskinesias. Keijsers *et al*.^19^, monitored 13 patients in a “home-like” situation, at the Occupational Therapy Department of the University Medical Center for 2.5 hours, using 6 accelerometers, located on different parts of the body. When they used the data from the whole sensor network they found good correlation with the outcome of the Abnormal Involuntary Movement Scale (AIMS). However, correlation between the scale and the different sensors individually was low (between 0.37 and 0.44); thus none of them alone was enough to detect the severity of dyskinesia. Devices based on multiple sensors distributed on the whole body are hard to use in clinical practice or for long monitoring periods; thus, our system has higher potential to be transferred to the clinical practice.

Hoff JI *et al*.^20^ conducted an experiment with 4 pairs of biaxial accelerometers, located on body segments of the side of the body, which was most affected by the Parkinson’s disease. As a comparison standard, they used video records and the Abnormal Involuntary Movement Scale (AIMS). They found good correlation between the sensor and the clinical scale (0.8–0.87), although only with the patient at rest or doing highly protocolized movements, and with sensors located on body segments that did not participate in movement. Thus, their results are also not applicable to patients’ daily life or to clinical practice. Similarly, Manson *et al*.^21^ found that patients with the highest AIMS scores had a larger frequency component in the 1–3 Hz band; however, they only measured during particular protocolized activities, in highly controlled situations; thus, their results do not seem to extrapolate to ambulatory measurement required in the clinical practice. Lopane *et al*.^22^ studied dyskinesia in 13 patients, who were standing still with eyes open, and found lower correlation than that described here, with their inertial sensors. Ramsperger R. *et al*.^23^ used gyroscopes to analyze the movement of 7 patients with leg dyskinesia at the laboratory and found low correlation with the Unified Dyskinesia Rating Scale (0.61). Although these authors completed the study with an experiment conducted in a real-world environment, they did not estimate the severity of dyskinesia in this part of the study, but just detected its occurrence. No further studies were found, where the severity of dyskinesia was estimated during natural patients’ activity outside the laboratory. Therefore, the work described in this paper is original in this regard. We postulate that inertial systems for symptom detection in Parkinson’s patients should be validated under real conditions of use, because the large amount of movements a person makes during daily life activity cannot be sufficiently studied through laboratory protocols or by assessing only predetermined movements of patients.

The algorithm used in this research, was earlier demonstrated to be valid for detection of dyskinesias; now, it is clear that its numerical output is also correlated with dyskinesia magnitude. However, the algorithm does not measure the occurrence of dyskinesias in a continuous way, but measurements are automatically discontinued if activities which interfere with detection occur (walking or changing position). This apparent limitation is actually a strong point, because it enables the disregarding of false-positive detections, when the sensor is used in real life conditions, with a huge variety of possible movements that can be mistaken for dyskinesias. Furthermore, the amount of data missed because of such an automatic discontinuation is often small, since most people, especially Parkinson’s patients, spend most daytime in relative rest, either standing up or sitting down.

Our study has some limitations such as the small tested-patient sample or the short monitoring time. It is hard to increase the monitoring time because, since the gold standard is a video record, it is hard to video-monitor patients for many hours. This might contribute to the scarcity of studies where dyskinesia is assessed with sensors and its severity is estimated (since this requires continuous observation). Such a short monitoring time is partly compensated by the fact that patients were asked to perform daily-life activities, in order to test the sensor.

The small sample size, similar to the other works discussed above, was sufficient to determine the high correlation in particular with the trunk and lower-limb UDysRS scores, within a reasonably narrow confidence interval.

Patients categorised as “mild dyskinesia of the limb” were excluded from the study; thus, the algorithm was not tested on such patients. Rather than a limitation of the algorithm, this is a limitation of the reference standard, because mild dyskinesia of the limbs is very hard to distinguish from voluntary movements or tremor, making comparison with the algorithm’s results unreliable.

In conclusion, the presented algorithm, which had previously shown good validity for detection of dyskinesia in real life conditions, does yield values that correlate with its severity and can therefore be useful to detect patients with severe dyskinesia. Given that the algorithm uses the signal from only one low energy inertial sensor, which can be comfortably worn on the waist, and given that it has been validated in real conditions of use, it has the potential to be used in clinical practice to help identify patients, who need therapy adjustment due to their dyskinesias.

## References

[1] Hughes AJ, Daniel SE, Kilford L, Lees AJ, Daniel SE (1992). Accuracy of clinical diagnosis of idiopathic Parkinson’s disease: a clinico-pathological study of 100 cases. Neurosurgery, and Psychiatry.

[2] Fahn S (2004). Levodopa and the Progression of Parkinson’s Disease. N. Engl. J. Med..

[3] Ahlskog JE, Muenter MD (2001). Frequency of levodopa-related dyskinesias and motor fluctuations as estimated from the cumulative literature. Mov. Disord..

[4] Fabbrini G, Brotchie JM, Grandas F, Nomoto M, Goetz CG (2007). Levodopa-induced dyskinesias. Mov. Disord..

[5] Papapetropoulos SS (2012). Patient Diaries As a Clinical Endpoint in Parkinson’s Disease Clinical Trials. CNS Neurosci. Ther..

[6] Rodríguez-Molinero A (2015). Validation of a portable device for mapping motor and gait disturbances in Parkinson’s disease. JMIR mHealth uHealth.

[7] Rodríguez-Molinero, A. *et al*. A kinematic sensor and algorithm to detect motor fluctuations in Parkinson disease: Validation study under real conditions of use. *J*. *Med*. *Internet Res*. **20** (2018). 10.2196/rehab.8335 PMC5943625 29695377

[8] Samà A., Pérez-López C., Rodríguez-Martín D., Català A., Moreno-Aróstegui J.M., Cabestany J., de Mingo E., Rodríguez-Molinero A. (2017). Estimating bradykinesia severity in Parkinson's disease by analysing gait through a waist-worn sensor. Computers in Biology and Medicine.

[9] Rodríguez-Martín Daniel, Samà Albert, Pérez-López Carlos, Català Andreu, Moreno Arostegui Joan M., Cabestany Joan, Bayés Àngels, Alcaine Sheila, Mestre Berta, Prats Anna, Crespo M. Cruz, Counihan Timothy J., Browne Patrick, Quinlan Leo R., ÓLaighin Gearóid, Sweeney Dean, Lewy Hadas, Azuri Joseph, Vainstein Gabriel, Annicchiarico Roberta, Costa Alberto, Rodríguez-Molinero Alejandro (2017). Home detection of freezing of gait using support vector machines through a single waist-worn triaxial accelerometer. PLOS ONE.

[10] Pérez-López, C. *et al*. Dopaminergic-induced dyskinesia assessment based on a single belt-worn accelerometer. *Artif*. *Intell*. *Med*. **67** (2016). 10.1016/j.artmed.2016.01.001 26831150

[11] Goetz CG, Nutt JG, Stebbins GT (2008). The Unified Dyskinesia Rating Scale: Presentation and clinimetric profile. Mov. Disord..

[12] Samà, A. *et al*. A double closed loop to enhance the quality of life of Parkinson’s Disease patients: REMPARK system. in *Studies in Health Technology and Informatics* **207** (2014). 25488217

[13] Rodríguez-Martín D, Pérez-López C, Samà A, Cabestany J, Català A (2013). A wearable inertial measurement unit for long-term monitoring in the dependency care area. Sensors (Basel)..

[14] *Parkinson’s Disease Management through ICT: The REMPARK Approach*. (River Publishers, 2017).

[15] Hoehn MM, Yahr MD (1967). Parkinsonism: onset, progression and mortality. Neurology.

[16] Giladi N (2009). Validation of the freezing of gait questionnaire in patients with Parkinson’s disease. Mov. Disord..

[17] Goetz CG (2008). Movement Disorder Society-Sponsored Revision of the Unified Parkinson’s Disease Rating Scale (MDS-UPDRS): Scale presentation and clinimetric testing results. Mov. Disord..

[18] Fisher RA (1915). Frequency Distribution of the Values of the Correlation Coefficient in Samples from an Indefinitely Large Population. Biometrika.

[19] Keijsers NLW, Horstink MWIM, Gielen SCAM (2003). Automatic assessment of levodopa-induced dyskinesias in daily life by neural networks. Mov. Disord..

[20] Hoff JI, van den Plas AA, Wagemans EA, van Hilten JJ (2001). Accelerometric assessment of levodopa-induced dyskinesias in Parkinson’s disease. Mov. Disord..

[21] Manson AJ (2000). An ambulatory dyskinesia monitor. J Neurol Neurosurg Psychiatry.

[22] Lopane G (2015). Dyskinesia detection and monitoring by a single sensor in patients with Parkinson’s disease. Mov. Disord..

[23] Ramsperger R (2016). Continuous leg dyskinesia assessment in Parkinson’s disease -clinical validity and ecological effect. Park. Relat. Disord..

